# The Histochemical Alterations of Mucin in Colorectal Carcinoma Quantified by Two Efficient Algorithms of Digital Image Analysis

**DOI:** 10.3390/ijms20184580

**Published:** 2019-09-16

**Authors:** Aldona Kasprzak, Agnieszka Seraszek-Jaros, Joanna Jagielska, Celina Helak-Łapaj, Elżbieta Siodła, Jacek Szmeja, Elżbieta Kaczmarek

**Affiliations:** 1 Department of Histology and Embryology, University of Medical Sciences, Swiecicki Street 6, 60-781 Poznan, Poland; ela_siodla@yahoo.com; 2 Department of Bioinformatics and Computational Biology, Chair of Pathomorphology and Clinical Immunology, Poznan University of Medical Sciences, Rokietnicka Street 4, 60-806 Poznan, Poland; agnetpa@gmail.com (A.S.-J.); jagielska@ump.edu.pl (J.J.); elka@ump.edu.pl (E.K.); 3 Clinical Eye Unit and Pediatric Ophtalmology Service, Heliodor Swiecicki University Hospital, Poznań University of Medical Sciences, Grunwaldzka Street 16/18, 60-788 Poznan, Poland; clapaj@ump.edu.pl; 4 Chair and Department of General Surgery, Endocrinological and Gastroenterological Oncology, Poznan University of Medical Sciences, Przybyszewski Street 49, 60-355 Poznan, Poland; jszmeja@ump.edu.pl

**Keywords:** colorectal cancer, mucin polysaccharides, PAS and alcian blue, histochemistry, digital image analysis

## Abstract

The practical use of knowledge on the diagnostic-prognostic role of polysaccharide components of mucins in colorectal cancer (CRC) has been difficult, due to the number of histochemical (HC) reaction types, as well as lack of standard methods of computer-assisted analysis of tissue expression of these molecules. Using two algorithms of digital image analysis (by application of Image-Pro Premier and our originally designed program Filter HSV), we evaluated the expression of polysaccharides in tissue samples of CRC patients (*n* = 33), and fragments of normal colorectal tissue from the same patients (control) using periodic acid Schiff reaction (PAS) (neutral mucins) and alcian blue staining (AB) (acidic mucins). Our results indicate lower expression of the PAS+ and AB+ mucins in CRC, as compared to the control samples. The higher expression of PAS+ polysaccharides was detected in flat tumors than in protruded CRC, while higher AB+ mucins expression was a feature of mucinous CRC subtypes. Positive correlation between mutual PAS+ and AB+ expression, as well as correlations with glucose concentration (PAS+ mucins), and hemoglobin level (AB+ mucins) were observed exclusively in unchanged colorectal samples (control). Both algorithms of digital image analysis (smart segmentation and Filter HSV) work properly and can be used interchangeably in daily practice of pathologists, as useful tools of quantitative evaluation of HC reaction in both normal and cancerous tissues.

## 1. Introduction 

Globally, colorectal carcinoma (CRC) is the third cancer in terms of incidence and second in terms of mortality [1]. In Poland CRC is the second most common cancer in men and woman, with the third leading causes of cancer deaths in Greater Poland Region [2]. CRC is characterized by high inter-patient and intra-tumor heterogeneity, as well as temporal molecular heterogeneity during treatment, which is known to influence the response to therapy and prognosis [3]. While genetic and epigenetic mechanisms are important and quite well described in colorectal carcinogenesis, the basis of the most cases of cancer is unknown. A small subset of CRC cases (up to 3%) arises as a consequence of inflammatory bowel diseases [4]. 

Both in histologically normal and morphologically changed colon, quantitative and qualitative changes in mucin polysaccharides were described for a few decades [5,6,7,8]. Some studies suggest that the aberrant and deregulated expression of mucins represent a link between cancer and inflammation [9].

Mucins are large *O*-glycoproteins with high carbohydrate content (over 70%). These mucin glycoconjugates provide the mucus with its biophysiochemical properties, due to their nature and extent of glycosylation [10]. Carbohydrates are critical for numerous biological processes (i.e., cell–cell adhesion, protein folding, protein trafficking, and cell signaling), with aberrant glycosylation implicated in a variety of diseases, including cancer [11].

During neoplastic progression, changes in the proportions of mucin glycoconjugates were described: e.g., neutral mucin increase, a reduction in the proportion of side chains of sialic acid, hypersecretion of weakly acid sialomucin and reduction of excessively acid sulfomucin [5,6,7,9]. Qualitative changes in sialic acid glycans include loss of O-acetyl substituents at C4, C7, C8, C9 or in the polyhydroxy side chain of sialomucin, causing some colonic tumors to contain much less mucin than the adjacent normal tissue [5,12,13]. 

Colon cancer mucins have differences in both core carbohydrates and peripheral carbohydrate structures that are being investigated as diagnostic and prognostic markers [14,15,16]. It is considered that CRCs producing large amounts of mucus indicate poorer prognosis [17], and mucinous subtype (~10–15% of cases) is a predictor of poor outcome [18,19,20]. 

Changes in tissue mucin carbohydrate content are most often analyzed with two basic histochemical (HC) techniques, namely periodic-acid Schiff (PAS) reaction, and alcian blue (AB) staining [13,16,21]. However, these methods are often modified: e.g., combined PAS/AB [8,9,16], mild PAS [6,22], PBT/KOH/PAS [7], PAT/KOH/PAS [12], KOH-AB pH 1-PAPS [23], HID-AB [7,8,24], and/or GOS sequence [25]. The large panel of techniques creates the need for evaluation of their ability to detect particular polysaccharide groups. Their ability to diagnose the largest possible amount of CRC patients (high degree of sensitivity), as well as identify people unaffected by the disease (high degree of specificity) is also very important [6,25,26]. It needs to be added that the progress of glycobiology is delayed by the difficulties in obtaining optimal, commercially available human glycan-specific antibodies used for immunohistochemical (IHC) reactions. To detect and monitor several cancers (including CRC), antibodies that target Sialyl Lewis A (Ca19.9) (antibodies to O-Glycans) are often used as serum biomarkers [11]. To evaluate the tissue polysaccharide expression, so called MUC1 specific glycan-Tn/STn-MUC1 was synthesized and used [27]. 

Traditional HC evaluation of mucin expression conducted by most of pathologists includes visual microscopic observation, use of general descriptions of HC reactions (color of staining reaction, positive/negative, weak, or no staining, etc.) [13,22], as well as the use of semi-quantitative scales for estimation of staining intensity and the proportion of positive cells [6,9,28,29]. The proportion of cells/tumors positive for a particular type of mucins is often indicated: neutral or acidic (sulfomucin, sialomucin) and mixed sialomucin and sulfomucin [6,29]. Due to staining heterogeneity of the CRC cells, the pathologists’ visual scoring and interpretation is highly subjective. 

The use of computer software to evaluate HC reaction in CRC can only be found in a limited number of studies. A prototype of such analyses could be the quantitative analysis of the area percentage of epithelium expressing galactose-oxidase-Schiff (GOS)-reactive saccharides in CRC, performed with the use of computer-assisted IBAS image cytometry system (AutoCyte, Inc. Elon College, Elon, NC, USA) [25]. Other authors evaluated deeply scanned mucinous CRC samples at x4 objective magnification with the use of NIS elements image analysis software (Nikon) [18]. Another study employed the Image J software and Adobe Photoshop, to complement the semi-quantitative analysis [29]. 

The number of technical modifications of HC reactions, evaluation of different regions of tumor (epithelium/complete mucosa), lack of unified tissue staining grading systems and difficulties in conduction of meta-analysis of changes in mucin glycoconjugates during colon carcinogenesis, motivated us to compare the results of analyses conducted with the use of two credible, fast and consistent computer-assisted techniques for HC evaluation of mucin expression in CRC. Protocol standardization and minimization of human input subjectivity is important in creating reproducible IHC and HC results.

The first aim of the study was to analyze the HC distribution of PAS- and AB-positive mucins in CRC and normal colorectal tissue samples (control), as well as to evaluate the diagnostic-prognostic role of the carbohydrates’ expression in colorectal carcinogenesis. The second aim was to show a clinical usefulness of two algorithms for quantitative evaluation of HC reaction in color microscopy images by using Image-Pro Premier and Filter HSV programs.

## 2. Results 

### 2.1. The Clinicopathological Features

The colonic adenocarcinoma was most often detected (*n* = 30), followed by adenocarcinoma recti (*n* = 3); one patient exhibited mixed neuroendocrine colonic adenocarcinoma with another sample recognized as adenocarcinoma in situ. Nonmucinous type was the most common CRC detected (70%). Ten patients were affected with adenocarcinoma of mucinous or partially mucinous subtype (30%). Sixteen patients had metastases to local lymph nodes, four patients had distal metastases in the liver. 

The clinicopathological features of the 33 patients with CRC at diagnosis are summarized in Table 1.

### 2.2. Detectability and Cellular Localization of Mucin Polysaccharide Histochemical Expression in Colorectal Carcinoma (CRC) and Control Samples

Using histochemistry, a positive PAS expression (magenta color) was detected in all CRC samples (100%) and in all control colorectal samples (100%). The expression of AB+ polysaccharides (blue color) was present in 94% of CRC samples and in all samples of the colorectal control (100%). 

**Figure 1:**
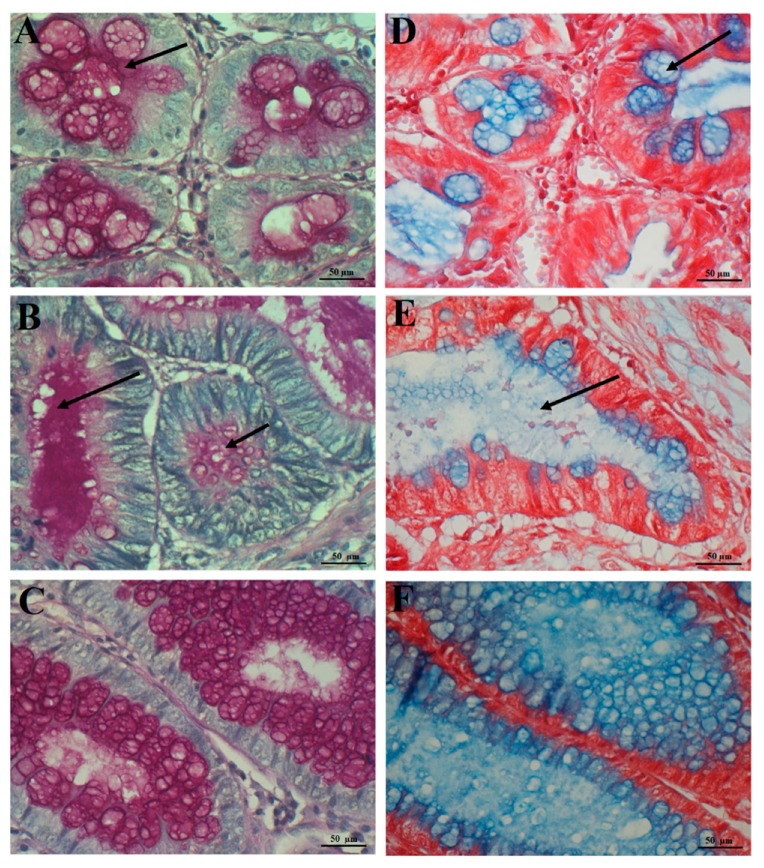
Illustrations with histochemical reaction in colorectal carcinoma (**A**,**B**,**D**,**E**) and normal colorectal tissue (control) (**C,F**). PAS reactivity in cytoplasm of the majority of neoplastic cells (arrow) (**A**); an intense PAS+ reaction in the extracellular mucus produced by neoplastic cells (arrow) (**B**); representative image of PAS expression in the control intestinal crypts (**C**); light blue AB staining in neoplastic cells (arrow) and in the extracellular mucus (arrow) of colorectal carcinoma specimens (**D**,**E**); dark blue AB staining in goblet cells and in the lumen of normal intestinal crypts (**F**). Hematoxylin (**A**,**B**,**C**) and safranin (**D**,**E**,**F**) counterstained.

PAS+ polysaccharides were located mainly in the cytoplasm of neoplastic epithelial cells of glandular structures of the tumor, as well as in the extracellular mucus (Figure 1A,B). Similarly, in control samples, proper color reaction was present in both the goblet cell cytoplasm and luminal part of the colorectal mucosa crypts (Figure 1C). Usually light blue acidic polysaccharides were observed in CRC structures (Figure 1D,E) and dark blue AB+ glycans in unaltered control tissue samples (Figure 1F).

### 2.3. Comparative Analysis of Results Obtained Using IRS and Two Computer-Based Methods 

**Figure 2:**
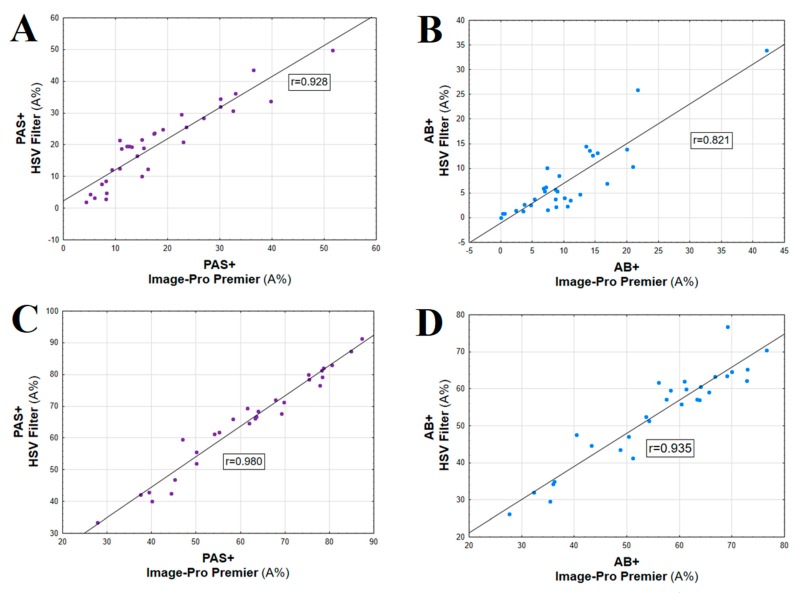
Correlation between two types of computerized methods used for quantitative evaluation of PAS+ and AB+ expression in colorectal carcinoma (**A** and **B**) and unaltered colorectal tissue samples (control) (**C** and **D**) (*p* < 0.05 in all cases).

Analysis of the results of PAS+ and AB+ expression in CRC and control samples obtained in semi-quantitative scoring system (IRS), and both computer-based methods showed high Cronbach’s alpha values and Spearman’s correlation coefficients (Figure 2A–D, Table 2). It needs to be noted that higher Cronbach’s alpha values concerned the comparison of two methods of computerized evaluation of HC reaction than the comparison of three methods (Table 2). High correlation between IRS scale vs. Image-Pro Premier for both reactions in CRC was found (*r* = 0.730, *p* < 0.01 for PAS and *r* = 0.778, *p* < 0.01 for AB). Slightly lower correlation was found between IRS scale and Filter HSV (*r* = 0.652, *p* < 0.02 for PAS and *r* = 0.665, *p* < 0.02 for AB) (data not shown). For all control tissue samples 12 points were assigned in IRS scale, therefore a correlation analysis was not performed. 

A comparison of polysaccharide expression in CRC and control tissue showed significantly lower expression of both PAS+ and AB+ polysaccharides in CRC tissues than in control colon samples from the same patients (Table 2). 

The results of quantitative analysis of PAS and AB reactions were well correlated with the use of smart segmentation method (Image-Pro Premier), in relation to Filter HSV (Figure 2). 

### 2.4. PAS+ and AB+ Polysaccharides Expression and Clinicopathological Features

#### 2.4.1. Mucinous vs. Nonmucinous CRC Subtype

**Figure 3:**
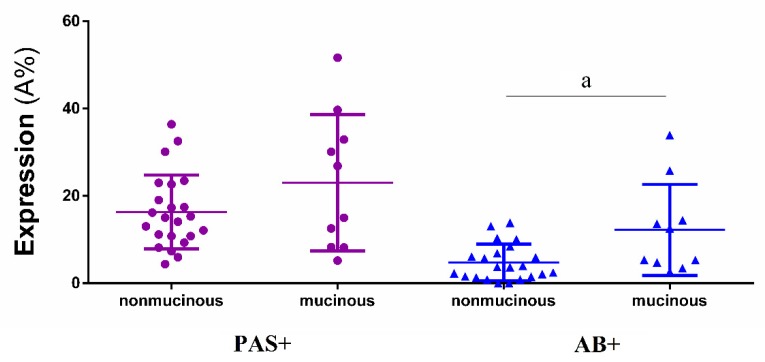
Comparative histochemical expression of PAS+ and AB+ polysaccharides (mean ± SD) in nonmucinous and mucinous subtypes of colorectal carcinoma. PAS+—periodic acid Schiff positive reactivity; AB+—alcian blue positive reactivity, a—significance = 0.028.

A significantly higher expression of AB+ polysaccharides was detected in the mucinous subtype, compared to other CRC subtypes (nonmucinous), when both Image-Pro Premier (Table 3, Figure 3) and Filter HSV (Table 3) were used, while differences in expression of PAS+ mucins between the two analyzed CRC subtypes were non-significant.

#### 2.4.2. Macroscopic CRC Type (Flat vs. Protruded)

Notably higher PAS+ polysaccharide expression was detected in flat tumors than in protruded, while non-significant differences were noted in expression of AB+ mucins (Table 4) independently on the computerized method of evaluation. 

#### 2.4.3. Localization of the Colorectal Tumor (Distal vs. Proximal)

No significant differences of PAS+ and AB+ polysaccharide expression (evaluated with the use of both computerized methods) were found between distal and proximal localization of the colon tumor (*p* > 0.05) (Table 4). It needs to be noted that using both computerized methods, in control colon of the same patients, higher expression of AB+ polysaccharides was detected in distal than in proximal region of the colon (*p* = 0.008), with similar PAS+ expression in both regions (*p* > 0.05) (data not shown). 

#### 2.4.4. Histological Grade of the Tumor

Non-significant differences of PAS+ or AB+ polysaccharide expression (evaluated with the use of both software types) were found between tumors of different levels of malignancy (G2 and G3 as most widely represented in CRC patients) (*p* > 0.05) (Table 5).

#### 2.4.5. Staging of the Tumor in TNM classification

No significant differences of PAS+ and AB+ polysaccharides expression (evaluated with the use of both computerized methods) were found in different TNM clinical stages (II vs. III as most widely represented in CRC patients) (*p* > 0.05) (Table 5).

### 2.5. Correlations between Mutual Expression of PAS+ and AB+ Polysaccharides in CRC and Control Colorectal Tissue 

**Figure 4:**
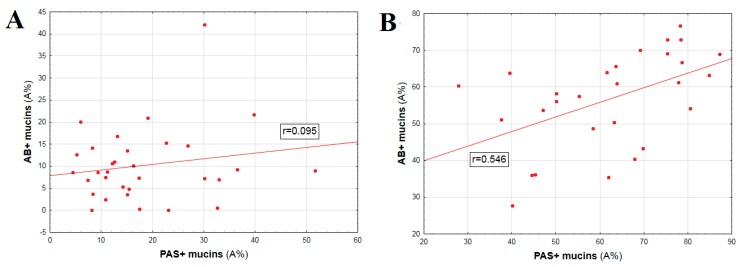
Correlation between mutual expression of PAS+ and AB+ polysaccharides in colorectal cancer *(p >* 0.05) (**A**) and unaltered colorectal tissue (control) (*p* < 0.05) (**B**); *r*—Spearman’s rank correlation coefficient.

In CRC samples this correlation was not significant (*r* = 0.095; *p* > 0.05). In turn, similar correlation reached statistical significance in unchanged colon samples (control) (*r* = 0.546; *p* < 0.05) (Figure 4). The results corresponded between two algorithms of digital image analysis. 

### 2.6. Expression of Polysaccharides and Selected Clinical Data 

Expression of PAS+ polysaccharides in CRC tissue samples was not significantly correlated with age and clinical data. However, PAS+ mucin expression evaluated in control samples showed positive correlation with glucose concentration, while AB+ mucins with hemoglobin level (*p* < 0.05) (Table 6). 

**Figure 5:**
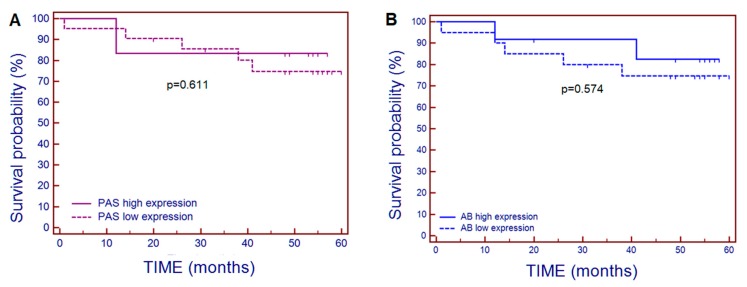
Kaplan-Meier survival curves for CRC patients related to tissue HC reactivity of PAS+ polysaccharides (**A**) and AB+ polysaccharides (**B**), showing that expression of both polysaccharide’s mucins in colorectal carcinoma tissue samples is not associated with survival time. High expression—above-mean tissue expression; low expression—below-mean tissue expression.

Mean survival time of patients affected by CRC in our study was 45.4 ± 16.5 months. The Kaplan-Meier analysis shows that neither PAS+, nor AB+ polysaccharides expression in tumor samples were significantly associated with survival probability of patients with CRC (Figure 5).

## 3. Discussion

In our study, there were no differences in PAS+ glycan detection frequency between CRC (100%) and control samples (100%). Other authors with the use of combined PAS-AB detection kit obtained ~70% positive tumors. However, they do not reference these results to control samples [16]. 

Quantitative data, obtained from two algorithms of digital image analysis, showed a decreased expression of both PAS+ and AB+ polysaccharides in CRC compared to “normal” colon samples. These results correspond with the results of other authors [29]. However, the information stating that with the increase of tumor grade, a considerable decrease in the acid mucin production and an increase in the neutral mucin expression is observed, cannot be confirmed [29]. Allen et al., using modified PAS reaction (PB-KOH-PAS), showed a significant diminution in the amount of O-acylated sialomucins in CRC, compared with its adjacent mucosa [7]. Similarly, Corfield et al. noted that total mucin sialic acid content significantly decreased with reduction of the O-acetyl transferase activity in CRC vs. control samples [30]. 

The results of studies that described an increase in the amount of neutral mucins, a reduction in the proportion of side chains of sialic acid [5] and/or hyper and moderate secretion of weakly acidic sialomucins in 88% of cases [9] cannot be confirmed, as particular functional groups of mucin glycoconjugates have not been analyzed. However, it seems that lower mean expression of both PAS+ and AB+ mucins detected in CRC samples, compared to control presented in our study, evidences proportional decrease of both these mucus polysaccharide types in colorectal tumors. 

We have not confirmed the reports of more intense staining of neutral (PAS+) polysaccharides in the right colon of normal colorectal region revealed by other authors [5,6], suggesting sialic acid heterogeneity within the general population [6]. However, we have observed increased AB staining in distal (left) vs. proximal (right) part of control colon. The difference in the presence of mucins between the left and right (more reactive for sialomucins) colons were shown in adenomas, since adenomas of the right colon are more protected by sialomucins and therefore less malignantly altered [31]. In our study higher PAS+ mucins expression was detected in flat tumors (<3 mm) than in protruded (height of tumor ≥3 mm) CRC. Other authors showed more reactive sulfomucins (HID-AB+) in adenomas of < 10 mm diameter [31]. 

The use of AB staining (pH 2.5) in this study allowed us to detect acidic mucins: sialomucins (dark blue), as well as sulfomucins (light blue), in 94% of CRC patients and in all of the control tissue samples. Other authors showed positive reaction for acidic polysaccharides in 60% CRC and only in 12% control samples [8]. Relating to the results in which a significant reduction in excessively acidic sulfomucins was observed [9], this study also noted a lower expression of AB+ polysaccharides in CRC vs. control. These results could confirm the suggested concept stating that a decline in the production of the sulfated mucins (sulfomucins) predisposes the colonic mucosa to malignancy [29]. 

Higher expression of AB+ glycans in mucinous CRC, compared to nonmucinous subtypes, finds confirmation in the literature [18]. In this study, no significant differences were noted in expression of PAS+ (neutral) and AB+ (acidic) polysaccharides depending on the different grade of CRC and clinical stages of cancer in TNM classification system. Other authors showed predominant cases with acidic mucins, especially in pure mucinous adenocarcinomas (>90%), while those with mixtures of acidic and neutral mucins were present in 62% of the cases. Clinical pure mucinous forms were detected mainly in advanced TNM stages [18]. Whereas, the present study showed a high positive correlation between mutual PAS+ and AB+ expression (*p* < 0.008), as well as correlations with glucose concentration (PAS+ mucins), and hemoglobin level (AB+ mucins), but exclusively in unchanged colorectal samples (control). The small number of dead patients (*n* = 7) analyzed in the current study, did not allow to draw binding conclusions on the predictive role of PAS+ and AB+ polysaccharides’ tissue expression for the survival time of patients with CRC of the Greater Poland Region. 

The two novel quantitative digital software types, used in this study, allowed for more objective determination of two types of mucin glycans’ expression, as well as correlation between the results of the analyses and clinicopathological data. Both Image-Pro Premier and Filter HSV can be used for evaluation of classic histochemistry results, as algorithms of initial microscopic picture analysis allow for appropriate counting of positively reacting structures. Image-Pro Premier is more accurate in counting faintly colored objects and objects located on uneven backgrounds, while Filter HSV is faster and more comfortable to use as a tool of HC reaction area in everyday pathological practice. IRS scale as evaluation system used in our study, is not suitable for assessing unchanged large intestinal mucosa tissues in which most cells produce mucus. These observations confirm the necessity of using more precise methods to quantify the expression of the mucins in healthy and cancerous tissues.

High correlation of quantitative expression results of two mucin glycans (PAS+ and AB+) was obtained with the use of smart segmentation algorithm and Filter HSV (Cronbach alpha value of 0.94–0.99). Both algorithms are highly reproducible. Both methods provide procedures of objective evaluation of HC reactions in pathological (CRC) and normal colon tissues (control). The polysaccharides expression using IRS scale correlated with expression obtained by using both algorithms of digital image analysis in CRC group. Comparing three methods (IRS, smart segmentation algorithm, and Filter HSV) for HC reaction evaluation, Cronbach’s alpha values were observed between 0.75 and 0.90.

Computerized analysis of tissue expression was better and more objective for precise evaluation of histochemical reactivity in case of PAS and AB than only semi-quantitative assessment using IRS score, especially in control colon tissue samples and/or in the cases of a very strong HC reaction.

The use of smart segmentation algorithm is accurate, particularly in the case of HC reactions that are difficult for credible visual evaluation, when the biomarker of interest is not uniformly distributed throughout the cell cytoplasm, or when it can only be found in the extracellular matrix. This algorithm also detects all (even faint) shades of magenta and blue. 

Overall, it could be concluded, that the smart segmentation seems to be more accurate in identification of faintly-colored objects and objects located on uneven backgrounds, while Filter HSV software is faster, easier to handle in computer-researcher interaction and more comfortable to use as a tool of HC are evaluation in daily practice of pathologists. 

## 4. Materials and Methods

### 4.1. Patients and Tissue Samples

Thirty-three patients (6 women and 27 men) were diagnosed and surgically treated between 2010–2015 (not treated previously with other forms of therapy), with the surgeries performed in the Chair and Department of General Surgery, Endocrinological and Gastroenterological Oncology, Poznan University of Medical Sciences. We have continued the study on selected patients with CRC only from the Greater Poland Region, from whom consent was obtained, and the perioperative tissue material met the requirements for scientific research [32]. Patients affected by diabetes, active chronic organ diseases, including autoimmune diseases and other cancers, have been excluded from the study. Inclusion criteria reduced the number of patients, but resulted in greater homogeneity of the group and reduced the potential impact of other factors on the investigated polysaccharides expression.

The patient ages ranged from 32 to 89 years (median of 67 years). Seven patients died during the analysis period. Duration of patient survival reflected the time between the date of CRC surgery and the initial diagnosis, i.e., 1 October 2010, and 1 October 2015. The available clinical data for CRC than was taken into account, included: descriptive histopathological diagnosis, histologic grade and stage (TNM classification), age, patient sex, and basic laboratory studies. The American Joint Committee on Cancer (AJCC) TNM clinical stages (seventh edition) [33] were assigned to each patient (0, I, II, III, and IV). 

Locations of the colorectal tumors were divided into proximal (right) colon and distal (left) colon (including rectum). Macroscopic types were divided into protruded type (height of tumor ≥3 mm) and flat type (height of tumor <3 mm). 

Thirty-three paired samples of colorectal tumor and control tissues were obtained simultaneously during surgical treatment. For the CRC, colon mucosa and, depending on the depth of tumor invasion, submucosal layers ~10–15 cm from the tumor site served as control tissues. In no case was tissue additional to that which would be removed normally during the usual surgical procedure taken from the patient. All tissue samples were fixed in 10% neutral buffered formalin for 24 h and routinely transferred into low-melting-point paraffin. To qualify the material for histochemistry, routine staining of the sections with hematoxylin and eosin (H+E) was performed. 

### 4.2. Histochemical Studies

#### 4.2.1. PAS (Periodic Acid Schiff) Reaction

Classic PAS reaction employing the Schiff reagent (decolorized/discolored fuchsin-sulphurous acid) was applied to qualitatively detect neutral mucin polysaccharides in epithelial mucins/mucosubstances, as it is negative for non-sulfated and sulfated acid mucins (sialomucin and sulfomucin, respectively) [6,21]. Paraffin sections on microscopic slides were dewaxed and immersed in growing concentrations of ethanol, 50%, 60%, 70%, 80% and 100%, for 5 min in each solution. Then, the samples were immersed in 1% periodic acid (HJO_4_)—5 min, washed twice for 2 min with distilled water, immersed in Schiff reagent for 30 min, washed thrice for 2 mins in sulfuric-acid water, washed under running water for 10 min and washed with distilled water. Finally, the paraffin sections were immersed for 2 min in hematoxylin, for counterstaining of cell nuclei, dehydrated in a series of alcohol solutions of increasing concentration and xylene, followed by placement under coverslips with the use of Canada balsam [5]. The Schiff reagent bound aldehyde groups, yielding purple/red (magenta) color [21]. The protocol of sulfuric-acid water (10 mL of 1 N HCl, 10 ml of 10% sodium metabisulfite water solution and 180 mL of water) and Schiff reagent preparation was in accordance with existing databases [34]. 

#### 4.2.2. Alcian Blue (AB) Staining

For selective detection of epithelial non-sulphated (sialomucins) and sulphated acid mucins (sulfomucins) (dark/light blue color, respectively) [21] alcian blue (AB) staining in pH 2.5 was applied (Sigma-Aldrich, Poznan, Poland). The subsequent stages included (1) deparaffination of slices; (2) incubation in 1% solution of alcian blue (pH 2.5) for 23 min; (3) washing under running water for 3 min; (4) incubation in 1% safranin O solution (Sigma-Aldrich, Poland) for 10 s; (5) washing under running water for 7 min; (6) fast dehydration in a serial alcohol dilution (<1 min) and slide mounting. It needs to be added that goblet cells of normal colon mucosa serve as a control for both HC reactions (PAS, AB) [6]. 

### 4.3. Semiquantitative Evaluation of the PAS+ and AB+ Mucins 

The PAS+ and AB+ polysaccharide expression in CRC and control tissue specimens were evaluated by the semi-quantitative 12-points score (immunoreactive score, IRS), originally designed to assess immunohistochemical reactions [35]. Number of cells with positive reaction (PP—percentage of positive cells) and intensity of the reaction (SI—staining intensity) were evaluated in 10 fields of Olympus B-2 microscope, at ×400 magnification. The final score reflected the product of the two variables (PPxSI) and ranged from 0 to 12 points (low reaction: 1 to 2 points, moderate reaction: 3 to 4 points, strong reaction: 6 to 12 points) and reflected the average number of PAS/AB-positive cells from all fields evaluated under microscope.

### 4.4. Quantitative Evaluation of Tissue Polysaccharide Expression

Histological slides with HC reaction were examined under the optical Olympus BH-2 microscope coupled to a digital camera. Color microscope images were recorded using a 40× magnification objective (at least 10 fields in every microscope slide with a PAS and AB-positive reaction) and LUCIA Image 5.0 computer software (2560 × 1920 pixels in size). A total of ~1300 color microscope images of CRC and control samples, stained with each of the used methods (PAS and AB), were separately counted using two algorithms. 

#### 4.4.1. Image Analysis by using Filter HSV Program 

**Figure 6:**
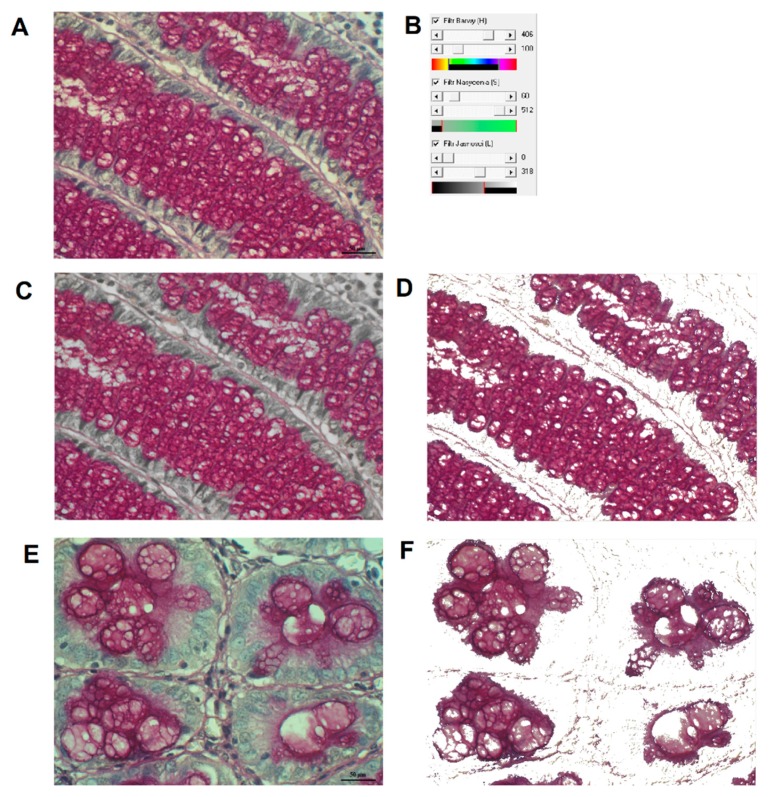
Typical steps for digital image analysis by using Filter HSV program concerning expression of PAS+ mucins in control colon (**A**) and colorectal cancer tissue samples (**E**). Row image (**A**); program settings for HSV space (**B**); successive stage of segmentation of PAS+ reaction (**C**,**D**); segmented PAS reaction (**D**); raw image (**E**); segmented PAS+ reaction (**F**).

The original Filter HSV program was worked out in the Department of Bioinformatics and Computational Biology, Poznan University of Medical Sciences [36,37]. In summary, for three coordinates of HSV color space: H (Hue), S (Saturation) and V (Value–brightness) ranges of colors (thresholds) specific for PAS and AB reactions were first assigned. Then, a segmentation of HC reaction was performed by thresholding (Figure 6). The program automatically counts pixels specific for the reaction and determines a percentage of HC reaction area, in relation to the tissue area, i.e., area fraction (A%):

A% = area of HC reaction (pixels)/tissue area (pixels) × 100%.

#### 4.4.2. Image Analysis with Smart Segmentation Algorithm by using Image-Pro Premier Software (Media Cybernetics v 9.3.2)

**Figure 7:**
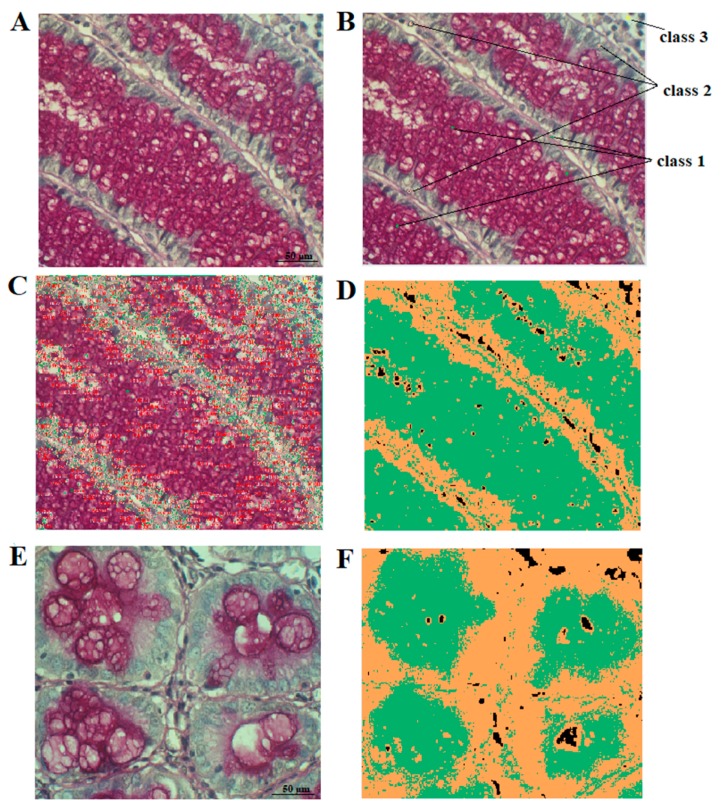
Typical steps for digital image analysis by using Image-Pro Premier software concerning expression of PAS+ mucins in control colon (**A**) and colorectal cancer tissue samples (**E**). Raw image (**A**); smart segmentation to identify PAS+ reaction—class 1, tissue area without reaction—class 2, background—class 3 (**B**); classification of structures (**C**); final PAS reaction image with mask applied (green color—PAS reaction, yellow—remaining tissue, black—background) (**D**,**F**).

Alternatively, a smart segmentation method was used, to extract and quantify the HC reaction. The method utilizes a pixel classification algorithm to identify objects and regions in three-steps. Thus, three classes of colors are defined (Figure 7):

- class 1: colors specific for the HC reaction,
- class 2: colors of tissue without the reaction,
- class 3: background (i.e., everything which does not belong to class 1 and 2).

After detection and interactive classification of colors specific for the reaction in given morphological structures, the number of pixels representing the reaction was counted and the percentage A% was computed according to the following formula: 

A% = (number of pixels belonging to class 1)/(number of pixels belonging to class 1 or class 2) × 100%. Ten microscopic fields were chosen randomly from samples of CRC tissue and the normal colorectal mucosa (control). In both methods, A% was determined in each of 10 fields per slide. 

### 4.5. Statistical Analysis 

Comparative analysis of results obtained using IRS scale and two computer-based methods were assessed by Cronbach’s alpha value. Two computer-based methods (smart segmentation algorithm and Filter HSV) were also assessed by Cronbach’s alpha coefficient. The consistency between the two computer-assisted techniques was confirmed by non-parametric Spearman’s correlation coefficient. All results were first verified by a normality test. Since the test confirmed a lack of normality, non-parametric methods were used for statistical analysis. Differences between PAS and AB expression in tumor tissue and in control were tested by the Wilcoxon test. Differences between unpaired results were verified by the Mann-Whitney test. 

Correlations between A% vs. age and clinical data were analyzed by the Spearman’s correlation coefficient. 

Two subgroups of tumor samples were also determined: below the mean expression and above the mean expression of PAS and AB reactions. The survival time of patients in both determined groups was analyzed by Kaplan-Meier and log-rank test. The statistical analysis was performed with Statistica v. 12. (Statsoft Inc., Tulsa, OK, USA). Results were accepted as significant at *p* < 0.05. 

### 4.6. Ethics Statement

Informed consent was obtained from every subject, with approval for the research obtained from the institutional bioethical committee on Bioethics, Poznan University of Medical Sciences, 61-701 Poznan, Poland (no. 924/14). The research protocol fulfils the standards recommended by the Helsinki Convention.

## 5. Conclusions 

Evaluation of histochemical expression of polysaccharides in CRC using digital image analysis, allowed to show that: (a) simultaneous, positively correlated PAS+ and AB+ polysaccharide expression in normal colon might indicate undisturbed production of carbohydrate mucin components in human; (b) colon carcinogenesis in humans is accompanied by significant decrease in tissue PAS+ and AB+ polysaccharide expression; (c) evaluation of HC reactivity in CRC allows to differentiate between the mucinous and nonmucinous subtypes; (d) both algorithms of digital image analysis (smart segmentation and Filter HSV) work properly and can be used interchangeably in daily practice of pathologists, as useful tools of quantitative evaluation of HC reaction in both normal and cancerous tissues.

## Figures and Tables

**Table 1:** Clinicopathological Characteristics of 33 Patients with Colorectal Carcinoma (CRC) at Diagnosis.

Characteristic		CRC (*n* = 33 Patients) (%)
**Age (yrs)**	<50	1 (3)
	≥50	32 (97)
**Sex**	male	27 (79)
	female	6 (21)
**Primary site**	Colon	30 (91)
	Rectum	3 (9)
**Gross morphology**	Protruded	20 (61)
	Flat	13 (39)
**Histologic grade (G)**	Carcinoma in situ	1 (3)
	G1	1 (3)
	G2	22 (67)
	G3	9 (27)
**Mucin content**	Nonmucinous	23 (70)
	Mucinous	10 (30)
**TNM stage**	Tis	1 (3)
	pT1-T2N0	3 (9)
	pT3-T4N0	13 (39)
	pT1-T2N1-N2	1 (3)
	pT3-T4N1-N2	15 (46)
	pM0	29 (88)
	pM1	4 (12)
	0 (Carcinoma in situ)	1 (3)
	I	3 (9)
	II	12 (36)
	III	13 (40)
	IV	4 (12)

G1—well-differentiated tumor; G2—moderately differentiated tumor; G3—poorly differentiated tumor; TNM staging according to the AJCC/UICC; Tis—carcinoma in situ; pT1 = tumor stage 1; pT2+ = tumor stage 2 or higher; N0 = lymph node stage 0; N1+ = lymph node stage 1 or higher; pM0 = distant metastasis stage 0; pM1 = distant metastasis stage 1), 0–IV—clinical stages of TNM system.

**Table 2:** Tissue expression of polysaccharides of both PAS+ and AB+ mucins in colorectal carcinoma (CRC) and in unaltered colorectal samples (control) evaluated by semi-quantitative score (IRS) and two types of digital software.

	Area Fraction of PAS+ [%]				Area Fraction of AB+ [%]			
	IRS	Image-Pro Premier	Filter HSV	*α*	IRS	Image-Pro Premier	Filter HSV	*α*
	[median]	[mean ± SD]	[mean ± SD]		[median]	[mean ± SD]	[mean ± SD]	
**CRC**	9.0	18.4 ± 11.3	20.4 ± 11.9	0.90 ^#^ 0.96 *	4.0	10.1 ± 8.3	7.1 ± 7.5	0.89 ^#^ 0.94 *
**control**	12.0	61.6 ± 15.9	65.1 ± 15.5	0.76 ^#^ 0.99 *	12.0	55.8 ± 13.5	53.1 ± 12.8	0.75 ^#^ 0.97 *
***p*-Value**	0.0003	<0.0001	<0.0001		<0.0001	<0.0001	<0.0001	

*p*-Value—comparing CRC and control; *α*—Cronbach’s alpha value between results from semi-quantitative scoring system (IRS), Image-Pro Premier and Filter HSV software (^#^); Cronbach’s alpha value between results of Image-Pro Premier and Filter HSV software (*).

**Table 3:** Comparison of PAS+ and AB+ polysaccharides expression in mucinous and nonmucinous subtypes of CRC (A%).

Subtype of CRC	Area Fraction of PAS+ [%]		Area Fraction of AB + [%]	
	Image-Pro Premier	Filter HSV	Image-Pro Premier	Filter HSV
	[mean ± SD] [median]	[mean ± SD] [median]	[mean ± SD] [median]	[mean ± SD] [median]
***mucinous***	23.1 ± 15.6	23.6 ± 15.8	14.9 ± 10.7	12.2 ± 10.5
	20.9	25.0	13.1	9.0
***nonmucinous***	16.4 ± 8.4	19.0 ± 9.9	8.0 ± 6.1	4.8 ± 4.2
	15.1	19.2	7.4	3.7
***p*-Value**	0.384	0.324	0.028	0.022

**Table 4:** Comparison of PAS+ and AB+ polysaccharides expression in flat/protruded macroscopic types of colorectal carcinoma, as well as proximal/distal localized CRC (A%).

Macroscopic type of CRC	Area Fraction of PAS+ [%]		Area Fraction of AB+ [%]	
	Image-Pro Premier	Filter HSV	Image-Pro Premier	Filter HSV
	[mean ± SD] [median]	[mean ± SD] [median]	[mean ± SD] [median]	[mean ± SD] [median]
***flat***	23.9 ± 12.6	26.5 ± 12.8	11.9 ± 11.1	8.3 ± 9.1
	22.7	24.7	9.0	5.4
***protruded***	14.8 ± 9.0	16.4 ± 9.6	8.9 ± 5.7	6.3 ± 6.3
	12.8	17.7	8.7	4.0
***p*-Value**	0.022	0.027	0.596	0.791
***proximal***	19.1 ± 9.3	21.5 ± 9.4	11.7 ± 12.9	8.8 ± 10.7
	19.0	21.5	7.5	6.0
***distal***	18.1 ± 12.2	19.9 ± 13.0	9.5 ± 6.0	6.4 ± 5.9
	15.1	18.9	8.7	4.8
***p*-Value**	0.630	0.475	0.934	0.869

**Table 5:** Tissue expression of PAS+ and AB+ polysaccharides in colorectal carcinoma as related to grading (G2 vs. G3) and TNM clinical staging according to the AJCC/UICC (II vs. III).

Grade/Clinical Stage	Area Fraction of PAS+ [%]		Area Fraction of AB + [%]	
	Image-Pro Premier	Filter HSV	Image-Pro Premier	Filter HSV
	[mean ± SD] [median]	[mean ± SD] [median]	[mean ± SD] [median]	[mean ± SD] [median]
**G2**	16.1 ± 8.1	20.0 ± 9.9	10.7 ± 9.2	7.0 ± 7.7
	14.6	19.5	8.7	3.8
**G3**	25.4 ± 15.8	23.5 ± 16.5	9.3 ± 7.2	7.9 ± 8.2
	26.9	28.4	9.0	5.4
***p*-Value**	0.188	0.453	0.894	0.885
**II**	17.1 ± 10.4	20.0 ± 12.0	7.6 ± 6.4	4.6 ± 4.3
	13.4	20.5	7.3	3.6
**III**	19.1 ± 9.2	20.9 ± 8.6	12.1 ± 11.3	9.9 ± 10.6
	15.4	20.9	8.7	6.3
***p*-Value**	0.406	0.810	0.347	0.242

**Table 6:** Values of Spearman’s coefficient for correlation between both types of polysaccharides expression (PAS+, AB+) in colorectal carcinoma (CRC), unaltered colorectal tissue samples (control) and selected clinical data.

Polysaccharide	Group	Digital Software	Age (yrs)	Hemoglobin (g/dL)	WBC (×10^9^/L)	Thrombocytes (G/L)	Glucose (mg/dL)
PAS+	CRC	I-P P	0.006	−0.017	−0.155	−0.015	−0.049
		HSV	−0.015	−0.063	−0.192	−0.008	0.072
	control	I-P P	0.007	−0.019	−0.054	−0.005	**0.401**
		HSV	−0.077	−0.042	−0.024	−0.043	**0.404**
AB+	CRC	I-P P	0.004	−0.208	−0.006	−0.062	0.225
		HSV	−0.019	−0.324	−0.161	0.038	0.113
	control	I-P P	−0.008	**0.457**	0.009	−0.251	−0.055
		HSV	0.029	**0.420**	−0.040	−0.216	−0.058

Bold numbers indicate values of significant r coefficient (*p* < 0.05); I-P P—Image-Pro Premier; HSV—HSV Filter.

## Abbreviations

AB	Alcian blue staining
AJCC	American Joint Committee on Cancer
CRC	Colorectal cancer
GOS	Galactose oxidase-Schiff’s
HC	Histochemistry
HID-AB	High Iron Diamine/Alcian Blue
HSV	H = hue; S = saturation; V = value (brightness)
IHC	Immunohistochemistry
IRS	Immunoreactive score
mPAS	Mild sodium periodate oxidation and Schiff
pAS (PAS)	Periodic acid Schiff reaction
PAT/KOH/PAS	Periodic acid/thionine Schiff/saponification/PAS
PBT/KOH/PAS	Periodic acid/borohydride reduction technique/saponification/periodic acid/Schiff
TNM	T = tumor; N = lymph nodes; M = metastasis
UICC	International Union for Cancer Control
